# Understanding rehabilitation and support needs after an episode of delirium: a qualitative thematic analysis of interviews with older people with delirium, family carers and healthcare professionals

**DOI:** 10.1186/s12877-025-06196-x

**Published:** 2025-07-26

**Authors:** S. Raghuraman, E. Richards, A. Mahmoud, S. Morgan-Trimmer, L. Clare, R. Anderson, VA Goodwin, L. Allan

**Affiliations:** 1 Department of Health and Community Sciences, University of Exeter, Exeter, England; 2 Royal Devon University Healthcare NHS Trust, Exeter, UK; 3 NIHR Applied Research Collaboration South‒West Peninsula, Bristol, England; 4 School of Primary Care, Population Sciences and Medical Education, University of Southampton, Southampton, UK

**Keywords:** Delirium, Delirium rehabilitation, Community rehabilitation, Qualitative research

## Abstract

**Background:**

Delirium is linked to adverse outcomes in older adults and independently increases the risk of developing or worsening dementia. While most research focuses on acute-phase management, long-term recovery remains poorly understood. This study explores post-discharge trajectories through a qualitative investigation of the perceived home rehabilitation and support needs of older adults following delirium.

**Methods:**

A thematic analysis approach was used to investigate the perceived home rehabilitation needs of older people who experienced delirium during hospital admission. Semistructured interviews were conducted with 46 key stakeholders (older people − 8, carers − 14, and healthcare professionals − 24).

**Results:**

Several interconnected themes were identified as potential resources that enable recovery from delirium: (a) social contact and recovery of social lives; (b) the need for information, support, and education; (c) personalisation and personhood; (d) relationship continuity with professional carers; (e) the experience and involvement of carers; and (f) treating the cause and healthy lifestyle.

**Conclusion:**

This study addresses notable research and practice gaps in the long-term treatment of delirium at home. This knowledge contributes to the development of an evidence-based theory of long-term delirium recovery.

**Supplementary Information:**

The online version contains supplementary material available at 10.1186/s12877-025-06196-x.

## Introduction

Delirium affects approximately 23% of older people in hospitals and is associated with a range of negative outcomes, including increased hospital stays, complications, distress, poor recovery, and higher mortality [1–4]. It is an independent risk factor for the development or progression of dementia [5–8] and can persist for up to 12 months in individuals with or without pre-existing cognitive impairment [9]. However, the pathophysiology of delirium remains poorly understood, and no effective medications currently exist for its prevention or treatment [10].

The societal and economic costs of delirium are significant. Poor recovery often necessitates increased levels of care or institutionalisation, leading to higher post-acute healthcare and societal cost [2, 11]. A systematic review estimated the global economic burden of delirium in 2019 to range from $806 to $24,509 USD (adjusted for inflation and currency) [12].

There is some evidence that that multicomponent preventive interventions can result in cost savings [13], and help prevent incident delirium in older patients [14]. Prevention strategies have largely focused on hospital and care home settings [15, 16],. with hospital-based programmes such as the Hospital Elder Life Programme (HELP) demonstrating cost-effective, nonpharmacological success in acute settings [17–20]. However, despite persistent symptoms after discharge and the ongoing burden on patients and caregivers, there is limited research into the treatment of delirium in post-acute, community settings.

Current clinical guidelines address the short-term management of delirium during hospitalisation, but there is a pressing need to build an evidence base for longer-term, community-based management to prevent further cognitive, functional, and physical decline [16, 21]. Rehabilitative interventions, which are genuinely person-centred, aim to restore function and reduce disability, offer a promising framework for post-acute delirium care [22]. In community contexts, this approach is often termed *reablement* and emphasises personalised, goal-oriented support [22]. Although evidence for delirium rehabilitation is limited, promising outcomes have been observed in populations with neurodegenerative conditions. Cognitive rehabilitation has proven effective in improving functional abilities and carer outcomes in people with mild to moderate dementia [23, 24]. while physical rehabilitation has shown positive results across all severities [25], including advanced stages [26, 27]. While dementia rehabilitation has a well-established evidence base and multicomponent rehabilitative interventions have shown promise for neurodegenerative conditions, such interventions for post-delirium recovery remain underexplored [28]. Dementia, being chronic and progressive, benefits from long-term cognitive and behavioural interventions, whereas delirium, an acute and reversible condition, requires rapid, targeted care. Existing dementia approaches do not address the acute cognitive fluctuations or hospital-related complications specific to delirium, highlighting the need for tailored delirium-specific rehabilitation. This gap justifies focusing on unique delirium recovery mechanisms, ensuring more effective interventions.

### Study background

This study is part of the RecoverED project, which aims to address this gap by developing and testing a novel, multicomponent, community-based intervention to support recovery after delirium in older people in the UK [29]. Following Medical Research Council guidance on developing and evaluating complex interventions [30], the RecoverED project uses a realist approach to understand what works, for whom, in what context, and why [31].

**Fig. 1:**
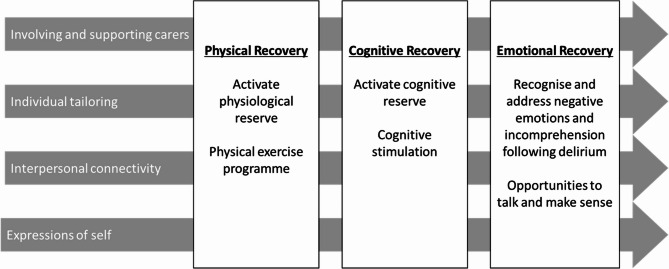
Preliminary programme theory of recovery from delirium ((28), p. 13)

An initial realist review identified three interrelated recovery domains and four facilitators, forming a preliminary programme theory (see Fig. 1) [28].

However, the study team concluded that additional specific components and their interactions are needed to achieve full recovery. Notably, many studies informing the review were not delirium-specific, drawing instead from wider populations such as individuals with dementia or ICU survivors.

This study aims to enhance understanding of relevant rehabilitation strategies to improve outcomes for older people recovering from delirium, forming part of the broader RecoverED initiative.

### Research questions

1. What are the perceived clinical and rehabilitative needs of older people after experiencing delirium?
2. What rehabilitative strategies might improve recovery from delirium after an episode?

## Study design and methods

The study has been reported according to the COREQ guidelines [32].

### Design

**Fig. 2:**
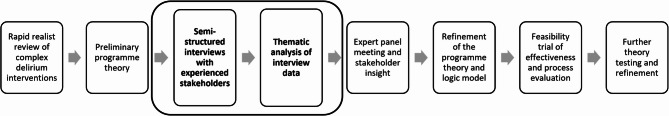
The RecoverED intervention development process

This study employed a qualitative thematic analysis (TA) approach to investigate what features of rehabilitation interventions are relevant to older people who have been discharged home after an episode of delirium. Figure 2 highlights the current study within the full process of the RecoverED project.

We sought the perceptions and views of health and social care professionals with experience in delirium diagnosis and care and older people with delirium and their carers.

### Ethics

This study was granted ethical approval by the Health Research Authority and Health and Care Research Wales (HCRW) (REC reference: 19/WM/0362). All the participants provided appropriate informed consent prior to their participation in the study in accordance with the Declaration of Helsinki.

### Patient and public involvement (PPI)

The views of a patient and public involvement (PPI) group played an important role in shaping the study. Their input informed the development of the topic guides, ensuring that questions were relevant, accessible, and sensitively phrased. They also advised on the format and language of participant-facing materials to enhance clarity and engagement. Additionally, the PPI group contributed to interpreting emerging findings, helping to ensure they reflected lived experience.

### Sampling

A purposive sample consisting of (1) healthcare professionals with experience treating delirium in hospital and community care settings (professional participants) and (2) individuals with experience with delirium (patient participants) and their informal/family carers (carer participants) was recruited from a large NHS Trust in Southwest England.

The inclusion criteria for patient participants were age over 65 years and recent experience of an episode of delirium diagnosed by the medical team in an acute inpatient setting. Eligible people either had the capacity to provide consent or had a nominated consultee who could consent on their behalf. Carers were included if they were family members or friends of the person with delirium who consented to participate and had the capacity to consent. Only English-speaking participants were included in the study.

A target sample size of 30 for each of the three groups was selected to provide data that were in depth and captured a range of experiences across the sample. Maximising variability was the main criterion of purposive sampling across a large spectrum of illness presentations and a wide range of contexts that were relevant to the intervention [33]. The sample size was determined based on the complexity of the phenomenon being studied and the variation within the sampling frame. As this study employed a partially deductive thematic analysis to develop programme theory, we did not use thematic saturation as a criterion for sample size determination, which is more appropriate for inductive qualitative methods such as grounded theory [34].

### Recruitment

Patient-participants with delirium were consecutively approached face-to-face during a period of admission to an acute medical or orthopaedic ward by research nurses. Carers who identified themselves as such were recruited with consent from patient participants. Written consent was obtained at the time of preparation for discharge. Patient and carer participants were eligible to be interviewed individually, but if both parties were available and accessible, they were interviewed together in patient‒carer dyads.

Professional participants were identified by consultation with the Dementia and Delirium steering group at the large NHS Trust in Southwest England, which is a group of clinicians responsible for improving dementia and delirium care in the trust. Purposive sampling was employed to invite people from different professional groups.

### Data collection

The participants took part in an in depth, semi structured telephone interview with either a trained and qualified research fellow (SR) or a junior doctor (ER) four weeks after discharge from the hospital. This timing was selected to allow a reasonable period for participants to begin recovering from delirium and have experiences of their needs for recovery while maintaining a reasonable period for the recall of their experiences. All interviews were conducted by telephone. When patient‒carer dyads were involved, they were interviewed jointly.

Two separate topic guides were used for the interviews, one for professionals and one for patients and carers (please see supplementary files 01 and 02). These were based on a previously conducted realist review of the literature and preliminary programme theory (28). Professionals were asked to reflect on existing care pathways for delirium, onwards services for people with delirium post-discharge, key components of a potential intervention for people with delirium, and delirium-specific training for healthcare professionals. People with delirium and their carers were asked about their experience of delirium after discharge, the services offered and utilised, any challenges faced in accessing services, their perspectives on recovery and what was needed in terms of services, and their thoughts on the preliminary programme theory. The interviews were audio recorded via an encrypted audio digital recorder, and consent to audio-recording was reconfirmed verbally at the time of the interviews. The completed interviews were analysed in parallel with the interviews conducted to revise the preliminary programme theory being tested and revise the subsequent interviews. This adheres to the iterative approach to programme theory development typical of realist studies [33, 35, 36].

### Data analysis

The audio recordings were transcribed verbatim for analysis, personally identifying information was redacted, and the transcripts were anonymised. NVivo 14 was used to manage the dataset.

**Fig. 3:**
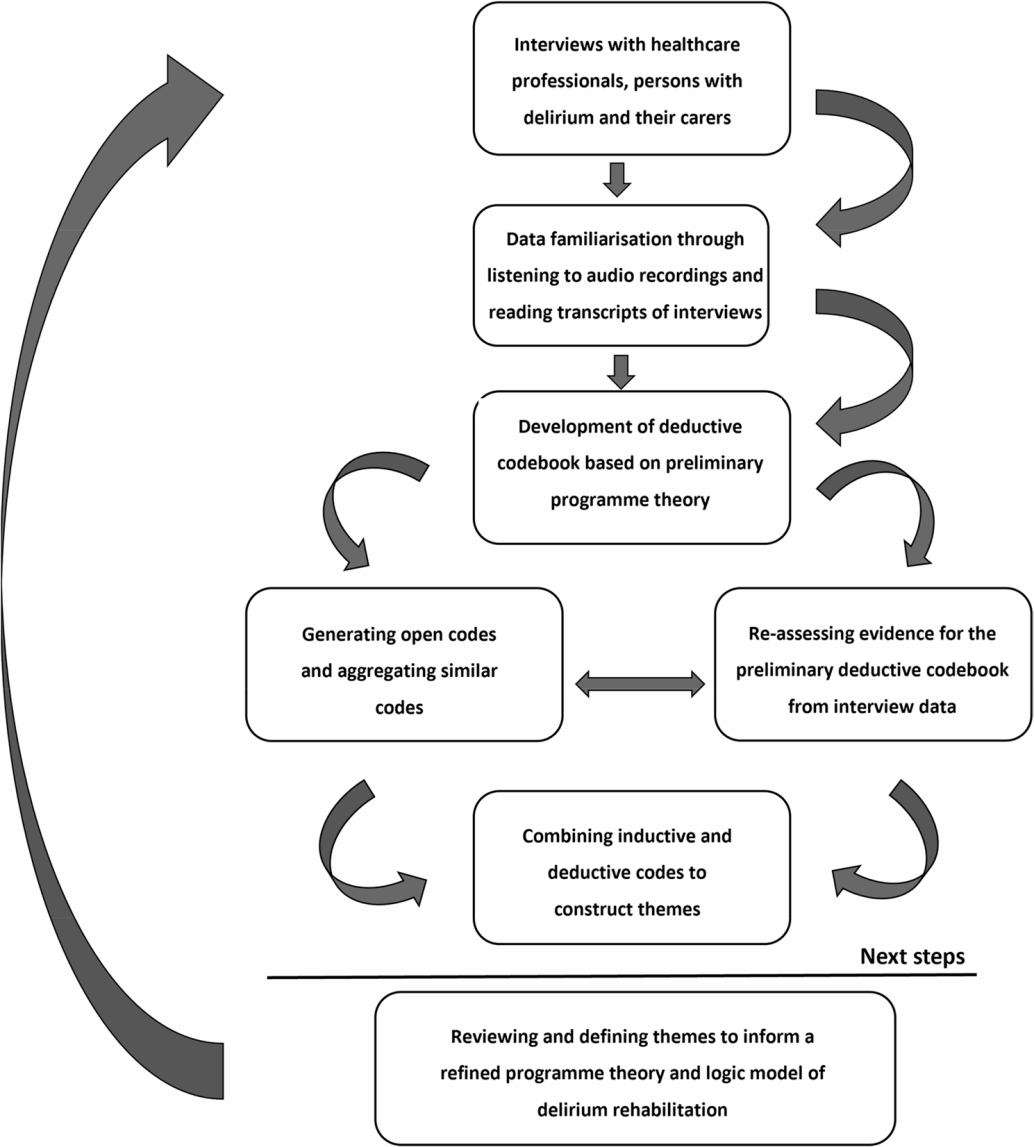
Stages involved in the modified thematic analysis

Reflexive TA was used to code and analyse the data owing to its epistemological independence and flexibility, making it suitable for health research [37, 38]. This adheres to the overall experiential and critical realist theoretical framework, which aims to understand participants’ lived experiences of delirium and recovery after delirium within the broader context of the project [39]. The authors SR and AM (trained research fellows) performed the initial coding and abstract theme development. Themes were then refined and reconfigured as necessary through discussions with the authors SMT (qualitative research expert) and LA (professor of geriatric medicine). The analytic approach drew deductively on the preliminary programme theory but also employed an inductive analysis to identify novel areas of interest and interpret them in relation to the research questions. The analytic process is illustrated in Fig. 3, with indications of the next stages of the RecoverED project following this study.

## Main findings

Forty-six interviews were conducted with older people with delirium (*N* = 8), their carers (*N* = 14), and health and social care professionals with delirium experience and expertise (*N* = 24). The sociodemographic details of all patient-participants who consented to participate (*N* = 17) are presented in Table 1. We were unable to interview nine patient-participants who initially consented for reasons related to ill health, lack of capacity, or being discharged to a facility that was not accessible via telephone.

**Table 1:** Sociodemographic details - Patient-participants

Age (in years)		M = 81.9, SD = 6.7	
Sex	Male		59% (*N* = 10)
	Female		41% (*N* = 7)
Ethnicity	White British		100% (*N* = 17)
Number of hospital admission days			M = 33.9, SD = 18.9
Discharge destination	Home		71% (*N* = 12)
	Residential care home		12% (*N* = 3)
	Community hospital		18% (*N* = 2)

*M* Mean, *SD* Standard Deviation, *N* Number

**Fig. 4:**
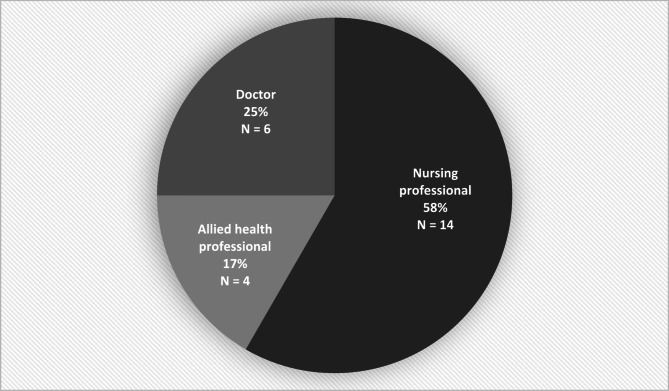
Categorisation of professional participants’ job roles

The final sample of professionals was both community- and hospital-based and had varying levels of experience and expertise in delirium care. Their professional roles are categorised in Fig. 4.

### Support for the preliminary programme theory

There was support from the interviews for the recovery domains and facilitators in the preliminary programme theory (Fig. 1). Professionals highlighted the value of repetitive, guided practice of cognitive and physical tasks as key mechanisms in promoting independent functioning in people with delirium. The participants also discussed the importance of staying physically active to improve mobility and reduce the risk of frailty and falls.

There is a general consensus that emotional support is necessary to address the psychological distress that could result from experiencing or witnessing an episode of delirium. There was also consistent support involving and supporting carers and individual tailoring of the intervention.

### Additional rehabilitation and support needs

**Fig. 5:**
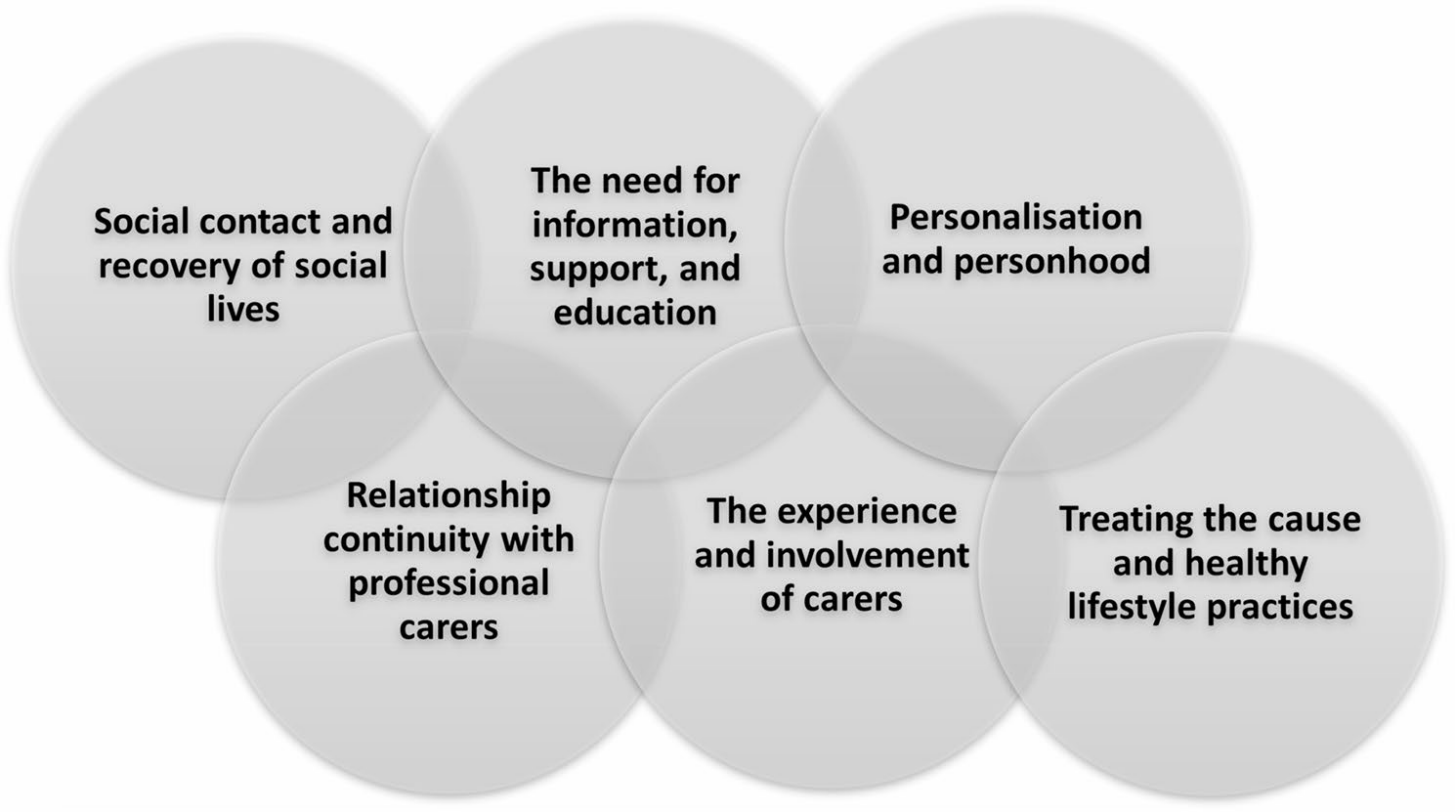
Interconnected themes related to recovery from delirium

Additional interconnected themes were identified that contributed to a deeper understanding of the resources and mechanisms of rehabilitation that could enable and support recovery within particular contexts to influence outcomes. These are presented across six broad themes (see Fig. 5).

#### Social contact and recovery of social lives

For some participants, experiencing delirium increased anxiety around social interactions, resulting in a lack of desire to engage socially.

*“What I have noticed is that her world has shrunk. So now she wants to spend time with me or me and my husband but doesn’t want it to be a bigger group. She’s very close to my son*,* and it was his 30th birthday. But it was an outing that she didn’t enjoy at all. Her anxiety levels were so high and that was because she didn’t have us just sitting beside her*,* making sure she was okay.”Carer P17.*

Some participants were eager to maintain social contact with a wider group during the episode of delirium and reiterated the potentially isolating experience of a hospital stay.

*“I think interaction socially with people is good. In the hospital ward… I was quite fortunate*,* there were other people there who were really quite interested in people*,* that had a good sense of humour. We had conversations and chatted.”Patient-participant P19.*

The experiences of isolation and motivation for social contact varied across the dataset, but many professionals felt that social reorientation was necessary for recovery to take place.

*“I can’t see how an elderly person would manage to get over delirium on their own… watching television day in*,* day out*,* being lonely and not having external prompts and external stimuli. you’ve got to have other people involved.”HCP13.*

They stressed the importance of taking into account what each individual needs in terms of social interaction.

*“We are social beings*,* so actually*,* how do people get back to what their experience of socialising is*,* what is normal for them? Is it that they would like to be able to work and be social in that respect? Is it that they want to be able to have meaningful outside activities*,* meet friends or is it that just once a week*,* they need to be able to have a coffee with their best friend?”HCP07.*

#### Relationship continuity in long-term community care

Carers highlighted the lack of one-on-one care or support during the hospital stay, which was sometimes attributed to constant ward changes or staff shortages. This heightened the care responsibilities of some carers, while others struggled to access timely information. These issues were exacerbated by poor follow-up and post discharge support in the longer term by community-based services such as the general practitioner (GP), leading to a lack of continuity of care.

*‘If a delirium is protracted and goes on for a long time at home… I like GPs to be picking that up a bit more from the discharge summary at least… [but] the GP doesn’t really see that patient enough to know what’s going on at home.’HCP02.*

Ensuring consistency in building positive relationships of care between the person, their family carers, and the professional was as important in addressing these issues.

*“… the continuity is really important because sometimes it’s quite an emotional thing*,* so you actually sort of need to really have a good bond with whoever’s coming to be able to sort of talk about it and trust them. And also*,* for the people doing the intervention to be able to see that it’s working or that things are going in the right trajectory.”HCP11.*

Relationship continuity could be a source of comfort for people with delirium, with trusted professional carers having a positive impact on some people.

*‘As far as [nurse’s name redacted] is concerned*,* she is number one*,* he doesn’t remember her name*,* but as soon as he saw her face*,* his face lit up on Monday when she came through*,* and there again*,* everybody is so good with him.’Carer P14.*

#### The need for information, support and education

Professional participants acknowledged the widespread prevalence of delirium, especially among older people, but felt that it was considered relatively uncommon by the wider medical community in the UK.

*‘There is a small group of professionals that might talk about a topic that actually impacts upon a huge proportion of patients. And I find that frustrating if I’m honest. It’s a lot of people and it affects a lot of patients*,* and yet it’s seen as a bit of a niche topic in a way.’HCP18.*

There appears to be a lack of awareness and clear training gaps among healthcare practitioners of all backgrounds regarding detection, diagnosis and appropriate referrals.

*‘The training need [is] massive. We are expecting the workforce to deal with really significant numbers of people with delirium*,* without any real training or knowledge of what they were doing.’HCP03.*

There was evidence from the interviews that suggested that people left the hospital with poor access to information about delirium from healthcare professionals.

*‘I would often see patients three or four days down the line and be the first person to say; your mum has delirium. And people would say*,* ‘Oh*,* really*,* what’s that?’ And actually*,* those conversations could be had a lot earlier*,* because patients and their families are left with an awful lot of anxiety because they don’t actually know what’s going on.’HCP03.*

Families often relied on other sources of information, such as relatives in the medical profession, the internet, or self-study, to understand what to expect and how to manage delirium at home.

*‘It’s scary if you don’t know what [delirium] is and I don’t feel that it was explained to us very much… because I didn’t know whether it was a permanent thing*,* whether it was going to last. I had to look it up myself rather than be told.’Carer P08.*

In terms of long-term care, many professionals highlighted the lack of a clear delirium care pathway in the community.

*“I wouldn’t say that there is a standard care pathway that’s used across our organisation*,* particularly for the community. I wouldn’t say that necessarily there’s a document*,* either electronic or written*,* which is used*,* and an automatic standard set of actions that are commenced.”HCP19.*

This resulted in families having to privately organise care plans for their loved ones with delirium.

*‘We tried all the normal sort of procedures… all the normal sort of channels and things*,* and in the end*,* my sister did a shout-out on Facebook and said*,* ‘Does anybody know who can take on someone for you know*,* private care? It was word-of-mouth and recommendation.’Carer P01.*

Most carers felt that access to educational support and signposting to resources at an early stage would be beneficial to people who have experienced a delirium episode.

*“Because it is quite frightening to a partner… somebody to sit down and talk to me and explain what was happening would have benefited me… because it scared me. More information… because I’d never heard of delirium before. The most important thing is explaining to the partner exactly what’s going on.”Carer P08.*

#### The experience and involvement of carers

The involvement of carers was identified as a key recovery facilitator in preliminary programme theory. This was based on studies that were situated in the hospital/acute setting or extrapolated from research on dementia care. The current study offers the value of extending family involvement in delirium care beyond the acute setting.

*‘Carers needs are that they need to be involved and communicated with right the way throughout*,* so from the point of admission to an in-patient unit right the way through. So*,* they need to be involved*,* there needs to be communication with the ward staff*,* dependent on what the patient wants.’HCP07.*

The value of actively seeking carers’ input was the opportunity to develop a long-term care plan that is tailored to the person’s needs, abilities and preferences.

*‘It is obviously very important to get a collateral history*,* so we’d call up carers or family to ask what the patient is usually like. We’d want to know whether they’ve potentially got undiagnosed dementia*,* so we’d also be getting collateral from that perspective.’HCP09.*

The analysis of the interviews provided a deeper understanding of the carer’s perspective. Witnessing an episode of delirium, illness progression or health deterioration was distressing to some carers.

*“It did stress us out the first few times actually to see him. He’s very… strong both mentally and physically*,* and to see him like that*,* like a child*,* it is scary… and very upsetting.”Carer P02.*

Ensuring that carers have access to support should also be a priority when considering their involvement in the care planning of people with delirium.

*“[Charity organisation] would phone me to see how things were going because the nurse got in touch with them and they contacted me; they wanted to know all about how I felt about things and what I could do. It was so helpful*,* absolutely helpful. It’s nice to be able to talk to somebody who understands the situation.”Carer P06.*

#### Personalisation and personhood

Individual tailoring was identified as an important recovery facilitator in preliminary programme theory and was strongly supported in this study. The analysis further builds on this by highlighting the value of goal setting as a suitable way of achieving personalisation and tailoring.

*‘It might be easier… for us to have discussions with the family about what do we really want as outcomes? And I think that every crisis within a [delirium] is an opportunity for us to reflect where are we*,* how is this going*,* what do we want*,* you know*,* what are our hopes and aspirations for this individual and their life in the future?’HCP17.*

This is closely related to upholding the individual’s personhood above aspects related to their delirium condition or illness status. According to one carer, their loved one struggled with changes in their environment that reinforced their illness status or loss of ability, suggesting that a sensitive approach to care and rehabilitation was necessary.

*“We sorted out a stairlift for him*,* and then*,* the subsequent week*,* we moved his bed downstairs. We couldn’t do it all in one go. He didn’t want the house to look like “an effing [sic] gymnasium” with all the medical paraphernalia everywhere*,* so we had to do it in small steps.”Carer P01.*

Supporting the person with regaining meaningful aspects of their life and focusing on their enduring abilities was identified as a positive approach to intervention.

*‘We try and understand them as a person… I [ask] the families [to] tell me what it is they love*,* is it gardening? Is it cars? I need something to work with. And when they’re really agitated*,* I’ll drop that into the conversation and just see if that little seed will develop into a conversation that’s about this place or where they are at the minute and not the world they think they’re living in.’HCP02.*

Recognising the value of empowering persons with delirium to regain independence through the guided practice of routine tasks was highlighted as a crucial approach to rehabilitation.

*‘I get a small amount of whatever they just said they liked in that cup and then help them by hooking their finger onto the cup. And they’ve got to do it themselves*,* but I will help them*,* and my hand will be around the cup with them so they don’t drop it and they don’t hurt themselves*,* but it is no good whatsoever for the nurse or the carer doing it for them*,* because their brain is not engaging in that activity. They have to be part of it for this to work*,*”HCP02.*

This follows a person-centred approach that advocates focusing on what the person *can do*, and which outcomes are most important to them, as opposed to the loss of abilities to motivate and encourage them to recover.

#### Treating the cause and healthy lifestyle practices

Treating the underlying physical or medical condition that could cause or exacerbate symptoms of delirium was also cited as necessary to treat delirium.

*‘All patients are exposed to the risk of delirium; we need a medical professional to assist in assessing what is the cause of it. So*,* if there’s an instability or insufficiency in minerals or vitamins in the bloods*,* is there infection? Is there something that we aren’t seeing? If we jump to try and manage the delirium*,* we may not be resolving the organ failure*,* [for example]’HCP14.*

Maintaining a healthy lifestyle was considered a necessary step toward preventing and managing long-term delirium. This could provide guidance for optimising diet, nutrition and hydration; reestablishing the sleep‒wake cycle; and normalising sleep routines.

*‘When a patient has a delirium*,* the focus collectively goes more to the medical cause and their physical health and wellbeing*,* so kind of within that occupational therapy*,* but are they eating? Are they drinking? Are they sleeping? All their physiological needs.’HCP14.*

Advice on optimising the home environment was found to be an important consideration when organising a home-based care plan. This is related to the belief that hospital environments are not optimised for recovery from delirium while also emphasising the need to make the home safe.

*‘Safety netting is can we alter the environment*,* not so much that it confuses them even more when they get home*,* but it makes it safer– for example*,* downstairs living.’HCP02.*

While not directly related to maintaining a healthy lifestyle, guidance on an optimal physical environment could help people recover in a safe, conducive environment.

## Discussion

This qualitative thematic analysis (TA) study sought to identify key features of rehabilitation strategies relevant to recovery after an episode of delirium in older adults. The findings validate and build upon our previous understanding of the domains and facilitators of delirium recovery, as outlined in a rapid realist review that included a broad search strategy but was not specific to post-acute delirium rehabilitation [28]. This TA extends the earlier review by incorporating a social dimension into the previously identified emotional component of delirium recovery, offering a more nuanced understanding of the complexities of social reintegration. Specifically, it highlights the role of relationship continuity with professional carers in facilitating this process, addressing a significant challenge in hospital settings: staff turnover, which can negatively impact recovery from delirium, a condition marked by confusion and cognitive impairments. Continuity of care is crucial for mitigating the adverse effects of staff disruptions on patients’ recovery trajectories.

Additionally, this analysis uncovers a critical gap not identified in the preliminary programme theory—the need for delirium-specific education. The study highlights that the lack of knowledge and support during and after discharge contributes to heightened anxiety and fear regarding home-based recovery. Furthermore, it extends our understanding of tailoring and personalisation, emphasising that interventions must be fundamentally person-centred. The findings also reinforce the importance of promoting a healthy lifestyle and treating underlying causes as essential components of delirium recovery.

Social contact and engagement emerged as key components of emotional recovery in the study. Structured activities that promote social interaction were viewed as beneficial for delirium recovery and for rebuilding confidence in functional tasks. Previous research has linked strong social networks to better cognitive function, a lower risk of functional decline, and reduced likelihood of postoperative delirium, emphasising the risks of social isolation [40–43]. In our study, restoring social connections was an important aspect of post-discharge rehabilitation. However, some individuals may face challenges in resuming their social lives. Long-term care plans should address personal preferences, needs, and barriers to social reintegration. To enhance clinical applicability, social contact could be actively supported through structured peer support groups or community-based activities designed to reduce isolation and promote emotional recovery. Moreover, the interpersonal relationship between a professional carer and a person with delirium can build confidence in social interactions. Relationship continuity could be facilitated by assigning a consistent care coordinator or single point of contact throughout the intervention, fostering trust and sustained engagement. Integrating these elements into service delivery models would address key psychosocial needs and enhance the effectiveness of post-delirium rehabilitation.

Strong evidence supports an educational component to inform and address the increased fear, anxiety, and loss of confidence experienced after delirium. Educational interventions for delirium have been shown to moderately increase confidence and competence, particularly in decision-making [44]. One study found that a low-cost educational intervention reduced delirium incidence and improved function in older medical patients [45]. Other studies have explored psychoeducation as part of multicomponent delirium interventions for carers [46]. A review of family involvement in delirium care suggested that enhancing carers’ understanding of delirium and its care has value, calling for more research into best practices for achieving this goal and evaluating their effectiveness [47].

Finally, the importance of maintaining a healthy lifestyle and addressing underlying medical conditions in the treatment of delirium is well established, both during episodes and in acute settings [20, 48–50]. We emphasise their ongoing relevance beyond acute care, particularly as components of community-based rehabilitation care plans, since many individuals experience persistent health complications following hospital discharge.

### Implications for practice

This study shifts the primary focus from preventing delirium incidence to rehabilitation outcomes such as enabling independence, functional recovery and autonomy and preventing further decline. There is currently no precedent for utilising multicomponent rehabilitation in post-acute delirium care. The novelty lies in the recommendation that the strategies identified in this research are all necessary, if not sufficient, and must be offered as part of an integrated, holistic care pathway, as opposed to being aimed at addressing symptoms in isolation or out of context.

Importantly, there is no evidence for effective rehabilitative interventions for delirium in the community setting. Once discharged, older people need support that is goal-oriented, is based on shared decision-making and aims at functional recovery in addition to symptom management. The professional participants in this study highlighted the benefits of home-based care for people with delirium, and the patient and carer participants shared their preferences for rehabilitation and recovery at home. It is unclear whether recovery is quicker or better after an episode of delirium at home than in the hospital. However, research has documented the high incidence and prevalence of delirium in intensive care unit patients, which is attributable to their exposure to a range of risk factors in acute settings [51]. Discharge to the home is also significantly associated with a reduced incidence of delirium [52], which suggests that home-based care could be better for recovery from and after delirium. This study highlighted inconsistencies in care pathways for patients with delirium, and there are risks associated with home-based care. Therefore, it is recommended that a well-planned and appropriately administered intervention can ensure that people can recover safely at home.

The study also highlighted training and knowledge gaps in delirium care, which has a knock-on effect on patients and carers. We found that delirium can often be missed, misdiagnosed, or managed poorly in the community. Supporting all healthcare staff with appropriate training, supervision and support with delirium management both in hospitals and in the community is crucial for supporting persons with delirium and their carers.

### Strengths and limitations

This study was conducted during the SARS-CoV-2 pandemic, which significantly limited recruitment, particularly of older people with delirium. The final patient-participant sample was smaller than intended, due to health, cognitive or communicative impairments that affected interview participation. Consequently, we relied more heavily on proxy view of carers, which may have excluded patients’ own perspectives.

The small interview cohort may also have introduced sampling bias by potentially excluding frailer individuals, those discharged to care facilities or those unable to participate due to cognitive or physical limitations, whose experiences and recovery needs may differ significantly.

The study may be subject to recall bias, as interviews were conducted four weeks after discharge, potentially affecting the accuracy of participants’ recollections; additionally, attrition bias may have influenced the findings, given that nine patients who initially consented did not complete the study.

The patient-participant sample included only individuals of White-British ethnicity. Minority ethnic groups remain underrepresented in healthcare research, and the exclusion of diverse voices limits our understanding of how cultural factors may influence healthcare use and responses to rehabilitation interventions [53, 54].

Furthermore, all participant groups were recruited from a single NHS organisation in Southwest England, where the demographics of older people may differ from those in other parts of the UK. As a result, certain themes related to delirium rehabilitation strategies may remain undiscovered due to the ethnic and geographic homogeneity of the sample.

Patient participants were recruited at admission or diagnosis, with interviews conducted four weeks post-discharge. As a result, the sample included individuals who had fully recovered by the time of interview and others who continued to experience symptoms, which highlights the difficulty of differentiating between recovery *from* delirium and recovery *after* delirium within this project [28]. Additionally, some participants were discharged home while others were transferred to care homes, which likely influenced the services they could access. These differences may have led to a conflation of diverse experiences of delirium presentation, symptomatology, and health status within a single theory of recovery.

The key strength of this study is its exploration of people’s perceptions of what is needed to affect recovery after delirium in post-acute settings. This study builds on our previous understanding of the mechanisms of recovery from delirium by investigating experiential accounts of delirium and what is needed for recovery after discharge. The themes constructed from these data bridge the gap between what is known about delirium rehabilitation in general and what additional features of rehabilitation are needed when planning a multicomponent home care plan for older people. This is a crucial step in the project’s overall realist approach to develop a programme theory of delirium rehabilitation in the community.

While the generalisability of our findings is limited by the single-site recruitment and specific patient profile, the theoretical depth achieved through thematic analysis and the development of programme theory, guided by a realist review, enhances the transferability of the findings.

In terms of reflexivity, the research team comprised individuals from mixed professional backgrounds, including three non-clinicians and two Research Fellows (SR and AM) with expertise in health services research and complex interventions. This mix of perspectives contributed to a more nuanced interpretation of the data by encouraging critical reflection and helping to mitigate individual biases. The involvement of non-clinical researchers also supported a broader consideration of psychosocial and contextual influences, ensuring that interpretations were not solely shaped by clinical perspectives.

### Future directions for research

This study aims to augment our limited understanding of the support and rehabilitation needs of older people with delirium after discharge from the hospital in the community setting. These findings contributed to the development of a programme theory, which served as the theoretical foundation for a novel, community-based delirium rehabilitation intervention [29]. This novel intervention addresses a lack of research on delirium treatment and management in post-acute settings.

## Conclusions

To conclude, this study addresses a significant gap in the long-term management of delirium in older adults within the community. It expands our understanding of the essential features of rehabilitation needed for an integrated, holistic care pathway. Notwithstanding the limited dataset on patient perspectives, this study contributes to the theoretical foundation for a novel rehabilitation strategy aimed at promoting functional recovery outcomes after delirium, with particular emphasis on supporting recovery in the community.

## Supplementary Information

Supplementary Material 1.

Supplementary Material 2.

## Data Availability

The datasets used and/or analysed during the current study are available from the corresponding author on reasonable request.
