# Supplementary material for: Understanding rehabilitation and support needs after an episode of delirium: a qualitative thematic analysis of interviews with older people with delirium, family carers and healthcare professionals
Source: BMC Geriatr. 2025 Jul 26;25:547. doi: 10.1186/s12877-025-06196-x (PMC12296708; doi:10.1186/s12877-025-06196-x)
Supplement: Supplementary file 1 — Supplementary Material 1. [file 12877_2025_6196_MOESM1_ESM.pdf]

Semi-structured interviews with health and social care professionals

Topic guide

Introduction

Introduce self and remind the respondent about the project

Remind them what delirium is

Explain purpose of the interviews: to explore a patient's (older person with delirium) needs after an episode of delirium, views of current service provision and identify suggested improvements for the future

Role and service

Can you tell me about your service and your role within wider NHS and other service provision?

Care pathways

Can you tell me about an example of a person with delirium you have seen and the problems they have had?

Can you tell me about the care / support pathway for people with delirium using your service?

Does the care / support pathway differ for people with dementia and people without dementia?

What are the key differences in providing care to people with delirium?

Is there anything you personally find particularly challenging about working with people with delirium? How do you address these challenges?

Onward services

Are patient s referred on to any delirium-specific services after an episode of delirium?

What other services would you use for people with delirium?

What criteria do you use when assessing which services to refer people on to?

Needs of people with delirium and their carers?

Do you think people who have experienced delirium in hospital have specific needs during their recovery once they have gone home from hospital? What are the key needs for recovery of people with delirium on going home from hospital?

Are people with delirium currently helped to recover from their episode of delirium after they go home? How well are carers of people with delirium supported?

Need for a specific service

Do you think it would be valuable to create a specific intervention for people with delirium and their carers? If so what should this look like? If not, why not?

Views on key components of an intervention for people with delirium

In terms of your own experience, can you give me an example of when a person with delirium recovered well and the factors which contributed to this? Can you give me an example of someone who did not recover well and the factors which contributed to this?

In an ideal world, how would a person with delirium be managed to maximise their recovery? Explore different needs at different stages in the illness trajectory (preparation for discharge, immediate needs on discharge, recovery over several weeks from discharge). What support should be provided for carers of people recovering from delirium?

What do you think should be included in a new service for people with delirium? Would some components be a higher priority than others?

Is there anything that should be in the intervention which is different for people who also have an underlying dementia?

From our research, we have identified some ideas for key components of an intervention, what are your thoughts on this?

- Discuss initial programme theories from the rapid review

Implementing change (facilitators and barriers)

How feasible would it be to deliver the new intervention in practice? What would be the key barriers? We will be piloting a new intervention, what suggestions do you have for overcoming these barriers?

Do you have any other ideas that would enable us to develop and deliver the new intervention?

Specific training needs in relation to people with delirium

Has your team had any training specific to delirium? What sort of training needs do you think your team has?

Is there any specific training that should be provided but isn't?

Measuring outcomes

How would you gauge the extent to which proposed interventions have been successful with people with delirium?

Would you recommend any formal tools for measuring outcomes? If so, which ones and why?
