# Supplementary material for: Understanding rehabilitation and support needs after an episode of delirium: a qualitative thematic analysis of interviews with older people with delirium, family carers and healthcare professionals
Source: BMC Geriatr. 2025 Jul 26;25:547. doi: 10.1186/s12877-025-06196-x (PMC12296708; doi:10.1186/s12877-025-06196-x)
Supplement: Supplementary file 2 — Supplementary Material 2. [file 12877_2025_6196_MOESM2_ESM.pdf]

Semi-structured interviews with older people with delirium and their carers

Topic guide

Introduction

Introduce self

My name is X and I am a researcher with the University of Exeter. We are trying to find out how best to help people recover after they have experienced delirium, like the one you experienced, and what we can do to improve things for other people in the future.

Why we're here

I'd like to talk to both of you about your experience of delirium in hospital and recovering from it since you have been home. We will also ask you about any help you have received since you have been home from both from family, friends and neighbours, and also help from healthcare professionals. We would also like to talk about your ideas for how we can improve in the future.

Remind them what delirium is

Delirium means different things to different people, and often means feeling confused or muddled, or feeling unable to concentrate on things. Some people may see or hear things that are unusual. Some people may also experience changes in their sleep, activity, and mood.

Process of the interview

This interview will last about an hour, but we can stop at any time. If you need a break you can have a break at any time. When we get to half an hour we will have a break.

Experience of Delirium

Core question: Could you start by telling me about your recent experience of delirium?

Follow-up/ prompt questions

- How did you feel in hospital?
- What sort of things did you experience (e.g. any abnormal experiences or beliefs)
- How long did your delirium persist?
- Which occurred in hospital and which at home? Trajectory (is it improving over time?)

To carer: Is there anything you would like to add from your perspective?

Experiences after coming home from hospital

Core question: Could you tell me how you have been managing since you got home from hospital?

Follow-up/ prompt questions

- Has the delirium persisted?
- What sort of things have you needed help with, if any? E.g. Help with personal activities of daily living (washing, dressing), help with instrumental activities of daily living (cooking, shopping, housework, using household appliances, medications, finances)

To carer: Is there anything you would like to add from your perspective?

Help received – Services used

Core question: Can you tell me about any professional help you have received as a result of the episode of delirium? For example, from doctors, social workers, nurses, voluntary organisations etc.

Follow-up questions:

- Who provided it? What did it entail?
- When was it? E.g. Immediately after discharge, within the next few days, any longer term support

Core question: Did it help you with the delirium?

- In what ways?
- What about it was particularly helpful?
- Context (home, hospital, day hospital etc)
- Duration and frequency (pacing, flexibility, whether perceived as enough, too much, too little)
- Content (relevance, easy to follow, right level of challenge, how tailored to individual)
- Interpersonal aspects
- Do you think this service helped you and if so how (prompt for specific impact, if any, on recovery from delirium)

To carer: Is there anything you would like to add from your perspective?

Help received – family and friends

Core question: Can you tell me about any support you have received from family/ friends/ neighbours following the delirium?

Follow-up questions:

- Who provided it?
- Specific help needed
- Duration and frequency
- Impact on family, friends or neighbours
- Impact for person with delirium (accepting help)

Core question: Did it help you to cope with the delirium?

- In what ways?
- What about it was particularly useful?

To carer: Is there anything you would like to add from your perspective?

Recovering from the episode of delirium

Core question: Have you recovered fully from the delirium you experienced or does it still affect you in some ways?

- What sort of things are you still experiencing?

Is there anything that might help you to recover more completely?

- Why would that be helpful?
- Within each aspect of life, can you give me some specific examples of the sorts of things you are thinking about?

To carer: is there anything you want to add from your perspective?

Improving services and developing a new approach for people who have experienced an episode of delirium

Core question: Thinking about the help you received after the episode of delirium, what was the best thing that we should make sure we include if we develop a new service?

Follow up questions:

- Explore needs at different stages in the trajectory and make sure ask about why they include each element and how they think it would be helpful/what impact it would have on the older person with delirium
- Anything else we should definitely include? Or not include?
- How could the help you received have been improved?
- Was there anything missing from the help you received that might have helped you make a better (or quicker) recovery?
- As well as the content, we need to make sure that any new service is easy for people to use. Was there anything that made it easier to use the help that was offered? Was there anything that made it more difficult to use the help that was offered or put you off using it?

Discuss initial programme theories from the rapid review

Core question: From our research, we have identified some ideas for key components of an intervention, what are your thoughts on this?

Close down

Is there anything else you'd like to say about your experiences or ideas for improving services?

To carer: Is there anything you would like to add from your perspective? Is there anything that we need to think about to make sure carers are supported as best as possible?
